# SERCA Modulators Reveal Distinct Signaling and Functional Roles of T Lymphocyte Ca^2+^ Stores

**DOI:** 10.3390/ijms252212095

**Published:** 2024-11-11

**Authors:** Md Nasim Uddin, David W. Thomas

**Affiliations:** Department of Pharmaceutical Sciences, Thomas J. Long School of Pharmacy University of the Pacific, Stockton, CA 95211, USA; m_uddin1@u.pacific.edu

**Keywords:** Ca^2+^ stores, SERCA regulation, ER Ca^2+^ pools, T cell activation

## Abstract

The allosteric SERCA (Sarcoplasmic/Endoplasmic Reticulum Ca^2+^-ATPase) activator CDN1163 has been recently added to the group of pharmacological tools for probing SERCA function. We chose to investigate the effects of the compound on T lymphocyte Ca^2+^ stores, using the well-described Jurkat T lymphocyte as a reliable cell system for Ca^2+^ signaling pathways. Our study identified the lowest concentrations of the SERCA inhibitors thapsigargin (TG) and 2,5-di-(*tert* butyl)-1,4-benzohydroquinone (tBHQ) capable of releasing Ca^2+^, permitting the differentiation of the TG-sensitive SERCA 2b Ca^2+^ store from the tBHQ-sensitive SERCA 3 Ca^2+^ store. We proceeded to test the effects of CDN1163 on Ca^2+^ stores, examining specific actions on the SERCA 2b and SERCA 3 Ca^2+^ pools using our low-dose SERCA blocker regimen. In contrast to previous work, we find CDN1163 exerts complex time-sensitive and SERCA isoform-specific actions on Ca^2+^ stores. Surprisingly, short-term exposure (0–30 min) to CDN1163 perturbs T cell Ca^2+^ stores by suppressing Ca^2+^ uptake with diminished Ca^2+^ release from the SERCA 2b-controlled store. Concomitantly, we find evidence for a SERCA-activating effect of CDN1163 on the SERCA-3 regulated store, given the observation of increased Ca^2+^ release inducible by low-dose tBHQ. Intriguingly, longer-term (>12 h) CDN1163 exposure reversed this pattern, with increased Ca^2+^ release from SERCA 2b-regulated pools yet decreased Ca^2+^ release responses from the tBHQ-sensitive SERCA 3 pool. Indeed, this remodeling of SERCA 2b Ca^2+^ stores with longer-term CDN1163 exposure also translated into the compound’s ability to protect Jurkat T lymphocytes from TG but not tBHQ-induced growth suppression.

## 1. Introduction

The T lymphocyte is the chief orchestrator of the adaptive immune system, coordinating and regulating the multi-level interactions and deployment of both cell-mediated immunity and antibody responses [1]. T cells initiate the critical early stages of an immune response via the signaling reactions induced by T cell receptor (TCR) binding to an antigenic stimulus. A major component of the antigen-activated TCR signaling pathway is an early rapid increase in cytosolic Ca^2+^ levels deriving from intracellular Ca^2+^ storage sites as well as activation of tightly coupled Ca^2+^ influx pathways [2,3,4]. T cell activation thus relies heavily on the functional integrity of intracellular Ca^2+^ stores, generally thought to reside in the endoplasmic reticulum (ER). Regulation and maintenance of ER Ca^2+^ levels is therefore essential for the activation of the TCR pathway, and the central family of ion-transporting enzymes mediating these functions are the sarcoplasmic/endoplasmic reticulum Ca^2+^-ATPases (SERCAs) [4,5,6,7]. The SERCA Ca^2+^-ATPases or Ca^2+^ pumps have attracted much interest as potential targets for drug modulation in disease states given their prominent role in contributing to Ca^2+^ release/uptake events integrated within the TCR-induced signaling framework [8,9,10,11,12,13,14,15,16].

In this study, we sought to further characterize the pharmacology of SERCA-regulated Ca^2+^ stores in T lymphocytes. There is a clear imperative to gain a better understanding of the roles played by Ca^2+^ stores as essential regulators of the complex spatiotemporal Ca^2+^ signal underlying critical early signaling events driving T cell activation [17,18,19]. A powerful approach to probing Ca^2+^ store functions in T cell signaling networks is to modulate SERCA pump function using an array of small-molecule pharmacological agents that can potentially provide a means for fine control of the various SERCA pump states as the primary regulators determining ER Ca^2+^ store levels [9,11,12,16,19].

Much has been learned about Ca^2+^ store regulation using the classic thapsigargin, cyclopiazonic acid, and 2,5-di-(*tert butyl*)-1,4-benzohydroquinone trio of SERCA blockers, clearly validating the profitable application of SERCA pharmacology in efforts to dissect Ca^2+^ signaling mechanisms [20,21,22,23]. Thus, to further augment our tools for SERCA pump modulation, there is a compelling interest to complement the SERCA inhibitors with compounds that can increase SERCA activity. We can potentially achieve a greater insight into the complex roles and functions of ER Ca^2+^ stores by utilizing pharmacological agents that can both downregulate as well as upregulate SERCA functional activity. At present, CDN1163 appears to be the best pharmacological agent with the capacity to increase SERCA activity, having been shown to exert a significant boost in SERCA enzymatic action in muscle and nonmuscle SERCA isoforms [8,12,15,19,24,25,26]. We were thus motivated to examine the effects of CDN1163 on Ca^2+^ stores in T cells, the critical central coordinator of the adaptive immune system with a uniquely pronounced dependency on ER Ca^2+^ stores and Ca^2+^ influx pathways underlying T cell activation.

## 2. Results

### 2.1. Low Concentrations of TG and tBHQ Induce Ca^2+^ Release in T Cells and Establish a Pharmacological Regimen for Specific Blockade of SERCA 2b and SERCA 3

Jurkat lymphocytes and rat splenocytes were loaded with Fura 2 to measure intracellular Ca^2+^ changes in response to SERCA drugs and other agents that mobilize cytoplasmic Ca^2+^. Experiments were conducted in the absence of extracellular Ca^2+^, except where indicated, to directly assess Ca^2+^ release events and Ca^2+^ store status without the need to consider Ca^2+^ elevation due to Ca^2+^ influx. To acquire additional information on Ca^2+^ release and Ca^2+^ store content as well as to utilize Ca^2+^ mobilizing agents impermeable to plasma membrane (PM), we also conducted experiments using Jurkat cells in permeabilized membrane assays.

In order to investigate the effects of the SERCA activator CDN1163 on T cell Ca^2+^ stores, we sought to characterize SERCA-regulated Ca^2+^ store function using Jurkat lymphocytes as the model system for T cell Ca^2+^ signaling [27,28,29,30,31].

Previous work has revealed that T lymphocytes express both SERCA 2b and SERCA 3 Ca^2+^ pump isoforms [32,33,34,35], yet it is not known what the specific function of these two SERCA pumps is with respect to regulating Ca^2+^ signaling events in T cell function.

**Figure 1:**
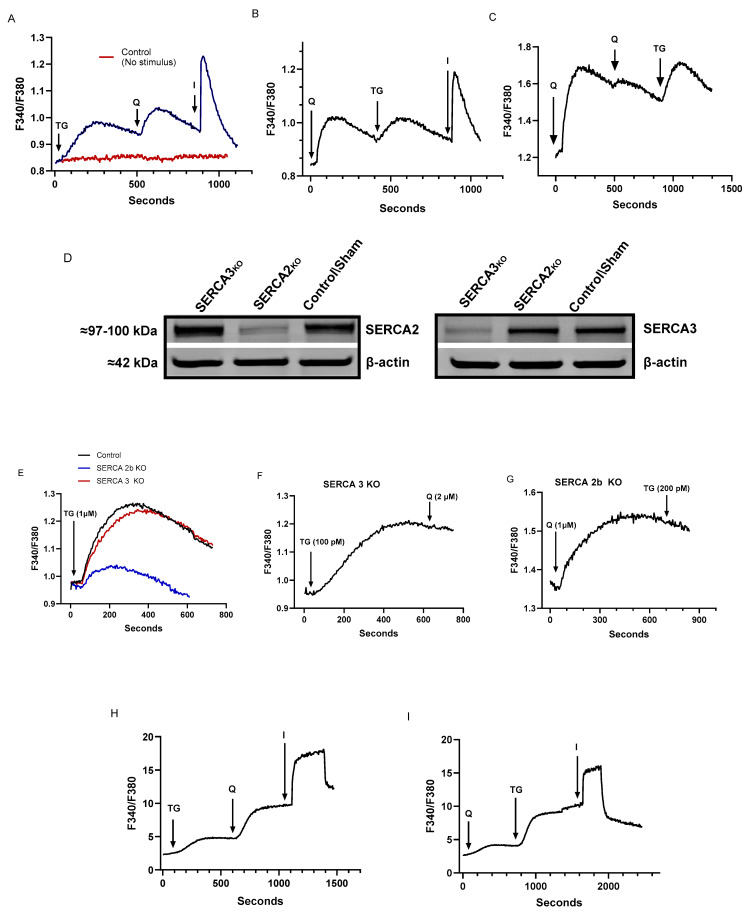
Low concentrations of the SERCA blockers thapsigargin (TG) and 2,5, di-(tert-butyl) 1,4-benzohydroquinone (tBHQ) can specifically induce Ca^2+^ release from distinct Ca^2+^ stores in Jurkat and rat T lymphocytes. For A and B, Jurkat T lymphocytes were loaded with Fura-2 and suspended in Ca^2+^-free media (balanced salt solution plus 2 mM EGTA). (**A**), Jurkat cell Ca^2+^ release responses induced by the sequential application (blue trace, arrows) of TG (100 pM), tBHQ (Q, 1 μM), and ionomycin (I, 1 μM) as determined by the ratio of fluorescence changes at 340 and 380 nm (F340/380). Red trace shows representative baseline fluorescence in absence of stimulus (**B**), the same experiment as in (**A**) but with the reverse application to Jurkat cells of tBHQ (Q, 1 μM), TG (100 pM), and ionomycin (I, μM). (**C**), the same experiment as in (**B**) but with consecutive tBHQ applications (Q, 2 μM) to deplete the tBHQ pool, followed by TG application (100 pM). (**D**), Western blot image of CRISPR-Cas9 knockout of the SERCA 2b and SERCA 3 Ca^2+^-ATPase isoforms in Jurkat lymphocytes (see methods). Figure shows the approximate molecular weight of the SERCA proteins and β-actin protein bands as controls. (**E**), SERCA 2b (blue trace) and SERCA 3 (red trace) knockout and control (black trace) Jurkat lymphocytes were loaded with Fura-2, and Ca^2+^ release responses were induced, as in (**A**), to TG (1 μM). (**F**), SERCA 3 knockout Jurkat lymphocyte Ca^2+^ responses were stimulated with TG (100 pM) followed by tBHQ (Q, 2 μM) as in (**A**). (**G**), SERCA 2b knockout Jurkat lymphocyte Ca^2+^ responses were stimulated with tBHQ (Q, 1 μM) followed by TG (200 pM) as in (**B**). (**H**), Rat spleen T cells loaded with Fura 2 and stimulated with TG (100 pM), tBHQ (Q, 1 μM), and ionomycin (I, 1 μM) in a balanced salt solution (HBSS, see methods) containing Ca^2+^. (**I**), Rat spleen T cells stimulated with tBHQ (Q, 1 μM), TG (100 pM), and ionomycin (I, 1 μM) in Ca^2+^-containing media (HBSS). Fluorescence traces shown are representative of four to ten separate experiments.

Figure 1 shows the establishment of the lowest concentrations of the SERCA inhibitors thapsigargin (TG) and 2,5-di-(*tert* butyl)-1,4-benzohydroquinone (tBHQ) capable of inducing detectable Ca^2+^ release responses in Jurkat lymphocytes with our methods. Figure 1A shows responses to the sequential application of TG (100 pM), tBHQ (1 μM), and the Ca^2+^ ionophore ionomycin (1 μM) in Jurkat cells loaded with Fura 2 and suspended in a Ca^2+^-free medium (also shown are representative baseline fluorescence signals in the absence of added stimulus). Following TG-induced Ca^2+^ release (ΔF340/380 = 0.16 ± 0.04 ratio units, *n* = 12), the further addition of low concentration tBHQ induces an additional increment of Ca^2+^ discharge on top of the elevated TG response (ΔF340/380 = 0.10 ± 0.007, *n* = 10) from intracellular stores, suggesting that the two SERCA blockers are acting on distinct Ca^2+^ storage sites. Lastly, the addition of ionomycin (1 μM) induces the release of remaining sequestered Ca^2+^ (ΔF340/380 = 0.28 ± 0.07, *n* = 12), suggesting that the releasable Ca^2+^ induced by low-dose TG and tBHQ is approximately half of the total Ca^2+^ stored in the Jurkat lymphocyte. Previous studies using a variety of hematopoietic-derived cells, including platelets and Jurkat lymphocytes, have revealed that low concentrations of TG specifically block SERCA 2b pumps, whereas low doses of tBHQ preferentially target the SERCA 3 isoform [32,36,37,38].

Figure 1B shows a similar experimental outcome where we reversed the order of application of the SERCA blockers, adding tBHQ first followed by TG addition. This result is consistent with the interpretation that the drugs used at these concentrations are revealing that the SERCA 2b and SERCA 3 regulated Ca^2+^ stores represent separate units of Ca^2+^ release in the Jurkat lymphocyte, given Ca^2+^ release responses are similar in magnitude (tBHQ: 0.18 ± 0.06, TG: 0.08 ± 0.003 ratio units, *n* = 6) and independent of the order of application of the SERCA blockers. We obtained additional evidence that the SERCA blockers are indeed releasing Ca^2+^ from distinct storage sites by depleting the tBHQ-releasable pool with higher tBHQ levels (2 μM), revealing that the TG-releasable store remains essentially unaffected in this sequence (Figure 1C, *n* = 4). As mentioned, there is a strong imperative to keep TG levels low in these experiments to maintain specificity for SERCA 2b blockade [32,36,37,38]. Thus, in contrast to Figure 1C, we could not effectively deplete the TG-releasable Ca^2+^ store without affecting tBHQ-induced release given that increasing TG levels inhibits SERCA3 pumps due to the general high potency for TG-induced inhibition on all SERCA isoforms. 

Indeed, we pursued this idea still further to verify isoform-specific targeting of the SERCA blockers by using CRISPR-Cas9 methods to edit expression levels of SERCA 2b and SERCA 3. Using this approach, we were able to knock out expression of SERCA 2b and SERCA 3 levels in Jurkat T cells (Figure 1D). We thus used the SERCA knockout cells to verify the specificity of TG and tBHQ by targeting the SERCA 2b and SERCA 3 Ca^2+^ pools, respectively. Figure 1E reveals that treating T cells with even high doses of TG (1 μM) largely abrogates Ca^2+^ release in SERCA 2b knockout cells, with the small residual responses likely due to release from SERCA 3 pools given the high concentration of TG. Moreover, Figure 1E shows that in the SERCA 3 knockout cells, TG responses are largely unaltered, consistent with TG’s actions to specifically target the SERCA 2b-regulated Ca^2+^ pools. Figure 1F,G further verify the SERCA blocker specificity. Figure 1F shows that the low-dose TG (100 pM) response is unaffected in SERCA 3 knockout cells, yet the subsequent addition of tBHQ (2 μM) fails to elicit a response (compared to Figure 1A) consistent with the absence of SERCA 3-regulated Ca^2+^ stores. Similarly, the tBHQ (1 μM) response is unaffected in SERCA 2b knockout lymphocytes while the subsequent addition of TG (200 pM), even at higher levels, fails to stimulate a further Ca^2+^ release response (Figure 1G), suggesting the absence of the SERCA 2b releasable pool (compare to Figure 1B).

Figure 1H,I shows Ca^2+^ responses to TG and tBHQ application in rat primary lymphocytes. As above, we used TG (100 pM) and tBHQ (1 μM) at low concentrations determined to work in Jurkat cell Ca^2+^ release experiments. We find that a similar effect of TG and tBHQ treatment is observed in primary lymphocytes, regardless of the order of drug application. We could not, however, perform these experiments in Ca^2+^-free media as the Ca^2+^ release responses induced by these low concentrations of TG and tBHQ were too small and variable to consistently measure in our experiments; thus, the experiments shown in Figure 1H,I reflect Ca^2+^ responses in primary lymphocytes suspended in Ca^2+^-containing media, and therefore we have reported initial peak height fluorescence changes that would be expected to capture the initial Ca^2+^ release response induced by application of the SERCA blockers. Previous work has reported sensitive communication and interdependency of ER Ca^2+^ release and the presence of external Ca^2+^ to support Ca^2+^ influx in T lymphocytes [28]. The Ca^2+^ responses activated by TG and tBHQ, albeit larger due to contributions from Ca^2+^ influx (Figure 1H, TG induced response: ΔF340/380 = 2.6 ± 0.62, *n* = 5; tBHQ induced response: ΔF340/380 = 5.2 ± 1.1, *n* = 5), suggest that primary lymphocytes contain the same Ca^2+^ pool profile as we observe in Jurkat lymphocytes with low concentrations of TG and tBHQ inducing release from SERCA 2b and SERCA 3 Ca^2+^ pools, respectively, and thus strengthen the validity of the Jurkat lymphocyte as a good model system for investigating SERCA regulation of T cell Ca^2+^ signaling networks.

### 2.2. Low-Dose SERCA Blockers and Agonist-Induced Ca^2+^ Release Patterns Suggest a Complex T Cell Ca^2+^ Pool Profile with up to Five Distinct Ca^2+^ Store Compartments

In order to investigate the effects of the SERCA activator CDN1163 on T cell Ca^2+^ stores, we sought to further characterize the properties and relationships of the SERCA 2b and SERCA3-regulated Ca^2+^ pools in Jurkat lymphocytes as revealed by treatment with low doses of TG and tBHQ.

**Figure 2:**
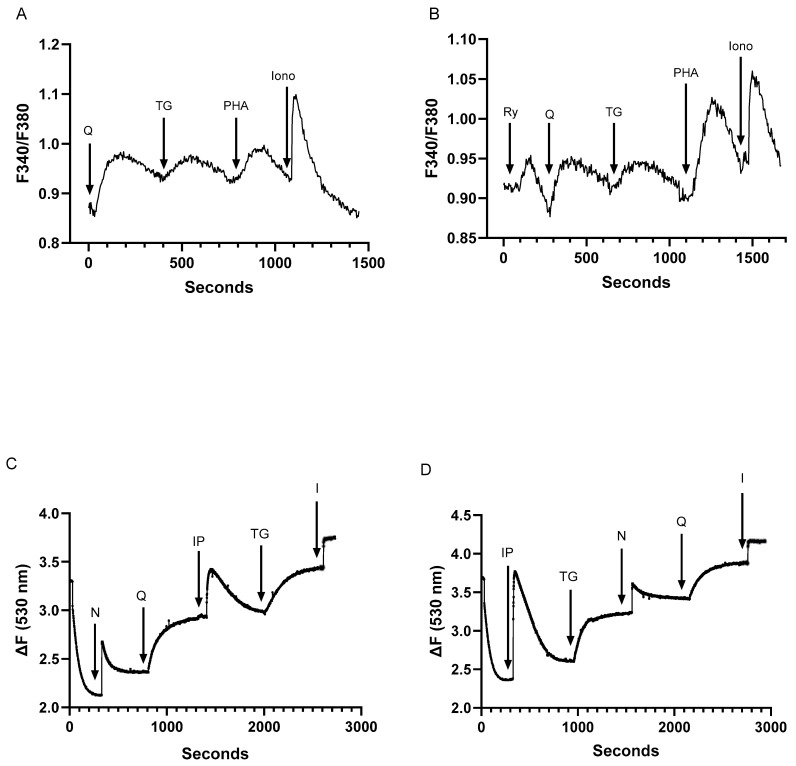
Low-dose SERCA blockers and Ca^2+^ release agonists reveal at least five distinct Ca^2+^ pools in intact and membrane-permeabilized Jurkat T lymphocytes. For (**A**,**B**), Jurkat T lymphocytes were loaded with Fura-2 and suspended in Ca^2+^-free media (balanced salt solution plus 2 mM EGTA). (**A**), Jurkat cell Ca^2+^ release responses induced by the sequential application (arrows) of tBHQ (Q, 1 μM), TG (100 pM), PHA (10 μg/mL), and ionomycin (I, 1 μM) as determined by the ratio of fluorescence changes at 340 and 380 nm (F340/380). (**B**), the same experiment as in A but with the sequential application to Jurkat cells of ryanodine (Ry, 30 μM), tBHQ (Q, 1 μM), TG (100 pM), PHA (10 μg/mL), and ionomycin (I, μM). (**C**,**D**) show experiments using saponin-permeabilized Jurkat cells suspended in an intracellular-like medium (ICM; see methods), depicting Ca^2+^ release responses as detected by Fuo-3 fluorescence changes. (**C**), permeabilized cell responses to the sequential addition of NAADP (N, 400 μM), tBHQ (Q, 1 μM), IP3 (IP, 0.5 μM), TG (1.5 nM), and ionomycin (I, 1 μM). (**D**). same experiment as shown in C but with the sequential addition of IP3 (IP, 0.5 μM), TG (1.5 nM), NAADP (N, 400 μM), tBHQ (Q, 1 μM), and ionomycin (I, 1 μM). Fluorescence traces shown are representative of six to ten individual experiments.

Previous work has identified at least four distinct Ca^2+^ stores in Jurkat lymphocytes, comprising inositol 1,4,5-trisphosphate (IP3), TG, ryanodine receptor (RyR), and ionomycin-releasable Ca^2+^ pools [27]. The recognition that low tBHQ concentrations release Ca^2+^ from a SERCA 3-regulated Ca^2+^ pool suggests one additional Ca^2+^ store and thus increases to at least five the number of distinct Ca^2+^ pools in the T lymphocyte Ca^2+^ signaling paradigm. Indeed, we show in Figure 2A the sequential release of Ca^2+^ induced by the application of low dose tBHQ (1 μM, 0.13 ± 0.03 ratio units) and TG (100 pM, 0.04 ± 0.008 ratio units), followed by a further Ca^2+^ release response induced by the addition of the T cell receptor crosslinker phytohemagglutinin A (PHA, 10 μg/mL, 0.07 ± 0.004 ratio units), which is known to mobilize Ca^2+^ from the IP3-sensitive stores [28]. Along with the ionomycin-releasable Ca^2+^ pool (1 μM, 0.17 ± 0.04 ratio units), Figure 2A suggests the presence of four intracellular Ca^2+^ pools in the Jurkat lymphocyte. Figure 2B shows that we can further induce Ca^2+^ release in this sequential application scheme by the inclusion of 30 μM ryanodine (0.04 ± 0.002 ratio units), revealing RyR, tBHQ, TG, PHA (IP3), and ionomycin releasable stores, thereby accounting for five distinct Ca^2+^ pools in the Jurkat lymphocyte. The final ionomycin-induced release in Figure 2B is approximately 35% reduced compared to Figure 2A (0.17 vs. 0.11 ratio units), consistent with the more extensive discharge of intracellular Ca^2+^ pools via application of both SERCA blockers and RyR/IP3R activators.

To examine the intracellular Ca^2+^ store profile in T lymphocytes more closely, we conducted experiments using permeabilized Jurkat T cells, allowing investigation of direct Ca^2+^ release responses induced by agonists impermeant to the plasma membrane. For these experiments, we used nicotinic acid adenine dinucleotide phosphate (NAADP) to release Ca^2+^ from RyR-sensitive stores and the direct application of IP3 to release Ca^2+^ from TCR (PHA)-coupled IP3R-sensitive stores in addition to the SERCA blockers and ionomycin. Figure 2C shows the detection of Ca^2+^ release from internal pools by the sequential application of NAADP (peak ΔF: 0.55 ± 0.03), tBHQ (ΔF: 0.59 ± 0.04), IP3 (ΔF: 0.49 ± 0.02), TG (ΔF: 0.46 ± 0.05), and ionomycin (ΔF: 0.31 ± 0.02). Figure 2D shows that regardless of the order of application, these Ca^2+^ release activators can induce release from five distinct intracellular storage sites. Thus, Figure 2C,D confirm the results observed from intact cell experiments, indicating the presence of five releasable Ca^2+^ pools in T lymphocytes.

### 2.3. Assessment of Inter-Relationships of Agonist Releasable Ca^2+^ Pools in T Lymphocytes

We explored the nature of the relationships between the SERCA 2b and SERCA 3 regulated Ca^2+^ stores to gain further insight into the functional roles of these distinct Ca^2+^ pools in T lymphocytes. Previous studies have reported that different Ca^2+^ mobilizing agents demonstrate the capacity to specifically induce Ca^2+^ release from SERCA 2b or SERCA 3 storage compartments, which may reveal recruitment of distinct Ca^2+^ release pathways corresponding to either SERCA 2b or SERCA 3 gated pools to subserve specific signaling functions in T cells, as has been shown previously for ADP secretion in platelets [39,40,41,42].

We chose to examine further a select group of Ca^2+^ mobilizing agonists that operate through the TCR, RyR, and GPCR Ca^2+^ release pathways. As above, we utilized both intact and permeabilized Ca^2+^ assays to determine the effects of SERCA 2b and SERCA 3 modulation on PHA, ryanodine, and thrombin responses in intact T lymphocytes and, correspondingly, IP3 and the RyR activator NAADP in permeablized cells.

**Figure 3:**
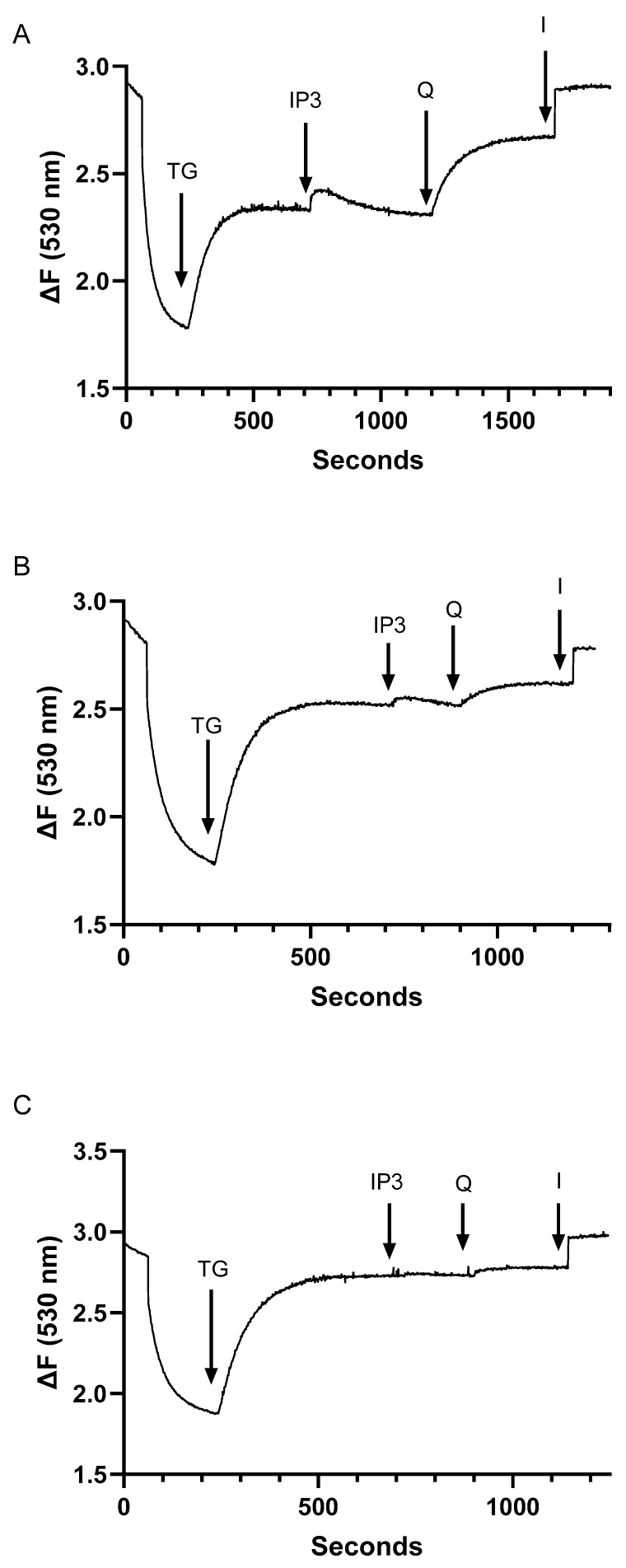
Increasing TG-induced SERCA inhibition depletes the IP3 and tBHQ-releasable Ca^2+^ pools, establishing the TG concentration range permitting SERCA pool-specific modulation in Jurkat T lymphocytes. (**A***–***C**) show experiments using saponin-permeabilized Jurkat cells suspended in an intracellular-like medium (ICM, see methods), depicting Ca^2+^ release responses as detected by Fuo-3 fluorescence changes. (**A**), permeabilized cell responses to the sequential addition of TG (2 nM), IP3 (0.5 μM), tBHQ (Q, 1 μM), and ionomycin (I, 1 μM). (**B**), Ca^2+^ release responses induced in permeabilized Jurkat lymphocytes by the sequential application of TG (10 nM), IP3 (0.5 μM), tBHQ (Q, 1 μM), and ionomycin (I, 1 μM). (**C**), Ca^2+^ release responses induced in permeabilized Jurkat lymphocytes by the sequential application of TG (15 nM), IP3 (0.5 μM), tBHQ (Q, 1 μM), and ionomycin (I, 1 μM). Fluorescence traces shown are representative of four to eight individual experiments.

In agreement with previous experiments using Jurkat lymphocytes [27], we find that the IP3-sensitive Ca^2+^ pool is a subcompartment of the larger TG-releasable Ca^2+^ store, given that we still observe TG (1.5 nM)-mediated Ca^2+^ release following IP3 (0.5 μM)-induced responses in permeabilized cells (Figure 2C,D). However, by adding TG first and gradually increasing its concentration up to just 15 nM, we can abolish the IP3-induced Ca^2+^ release responses (Figure 3A–C), verifying that with more aggressive SERCA 2b inhibition, we deplete the IP3-sensitive Ca^2+^ pool. Indeed, by increasing TG levels to 15 nM, we find that we can abolish tBHQ responses as well, indicating loss of the ability to pharmacologically discriminate between SERCA 2b and SERCA 3-regulated stores at this TG concentration (Figure 3C). These observations provide assurance we can perturb TCR-coupled IP3-sensitive Ca^2+^ stores via specific pharmacologic modulation of SERCA 2b function with low dose TG.

**Figure 4:**
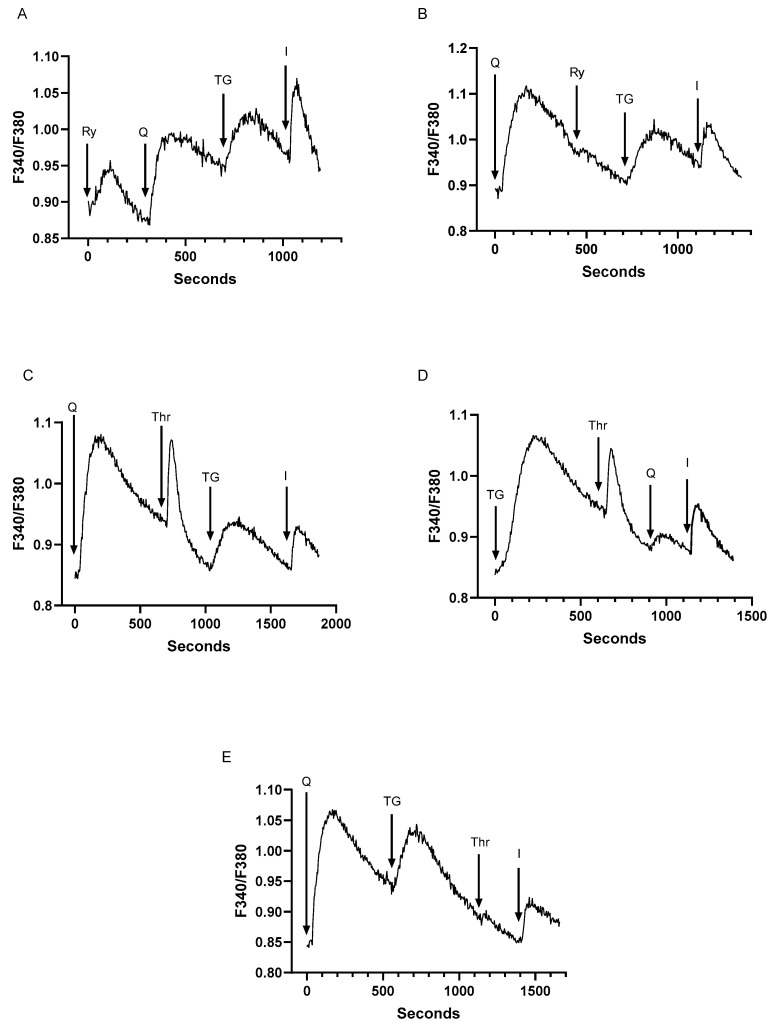
Relationships of the Ryanodine and Thrombin releasable Ca^2+^ pools to the low dose TG SERCA 2b and low dose tBHQ SERCA 3 regulated Ca^2+^ stores. For (**A**–**E**), Jurkat T lymphocytes were loaded with Fura-2 and suspended in Ca^2+^-free media (balanced salt solution plus 2 mM EGTA). (**A**), Jurkat cell Ca^2+^ release responses induced by the sequential application (arrows) of ryanodine (Ry, 30 μM), tBHQ (Q, 2 μM), TG (200 pM), and ionomycin (I, 1 μM) as determined by the ratio of fluorescence changes at 340 and 380 nm (F340/380). (**B**), the same experiment as in (A but with the sequential application to Jurkat cells of tBHQ (Q, 2 μM), ryanodine (Ry, 30 μM), TG (200 pM), and ionomycin (I, μM). (**C**), Ca^2+^ release responses to the sequential addition of tBHQ (Q, 2 μM), thrombin (Thr, 0.1 U/mL), TG (200 pM), and ionomycin (1 μM). (**D**), Ca^2+^ release responses to the sequential addition of TG (200 pM), thrombin (Thr, 0.1 U/mL), tBHQ (Q, 2 μM), and ionomycin (1 μM). (**E**), Ca^2+^ release responses to the sequential addition of tBHQ (Q, 2 μM), TG (200 pM), thrombin (Thr, 0.1 U/mL), and ionomycin (1 μM). Fluorescence traces shown are representative of four to seven individual experiments.

RyR signaling in T cells has been marked by a relatively high degree of irresolution given mostly low expression levels of the receptor in lymphocytes [43,44,45]. Nonetheless, recent work claims a prominent role for RyR in shaping the earliest Ca^2+^ signals essential for T cell activation [43,44,46]. Jurkat T lymphocytes as a clonal homogeneous population have been useful in clarifying the roles of RyR in T cell signaling given their expression of RyRs, albeit at low levels [28,47,48]. Studies using Jurkat lymphocytes have revealed that the Ca^2+^ mobilizing agonist NAADP acts on RyRs to induce Ca^2+^ release from the ER and not from a unique, separate acidic Ca^2+^ store compartment as has been observed in platelets [49,50]. Moreover, platelet studies have indicated that the SERCA 3 Ca^2+^ pump and not the SERCA 2b isoform controls the NAADP Ca^2+^ releasable store [39,51]. Thus, we performed experiments to determine whether T cell RyR-regulated Ca^2+^ stores, like platelet NAADP releasable pools, are affiliated specifically with SERCA 3 pumps using our low-dose TG and tBHQ SERCA blocker regimen. Figure 4A shows that ryanodine (30 μM) induces a small Ca^2+^ transient (0.06 ± 0.005 ratio units, *n* = 5) in Jurkat cells suspended in a Ca^2+^ free medium, which rapidly decays in the presence of functional SERCA 2b/3 Ca^2+^ pump activity. The ryanodine-inducible responses have no effect on subsequent Ca^2+^ release responses induced by tBHQ (0.12 ± 0.008 ratio units) and TG (0.08 ± 0.006 ratio units), suggesting that RyR activation in the Jurkat lymphocyte is inducing release from a smaller subcompartment of the larger tBHQ and/or TG releasable pools. However, when we reverse the order of application and increase the concentration of tBHQ, we find that we can abolish the ryanodine-inducible response (Figure 4B, *p* < 0.05, *n* = 5) with little effect on the subsequent TG-induced Ca^2+^ release response (Figure 4B). This finding suggests that, similar to platelets, NAADP releasable Ca^2+^ stores are replenished by SERCA 3 Ca^2+^ pumps in T cells.

We investigated whether we could alter thrombin responses in T cells using low-dose TG and tBHQ to establish a specific linkage of thrombin-releasable Ca^2+^ stores with SERCA 2b or SERCA 3 Ca^2+^ pools. Thrombin acts on G protein-coupled receptors (GPCRs) and has been observed in platelet studies to be coupled to two distinct signaling functions [40,41]: thrombin-induced Ca^2+^ release from SERCA 3-regulated pools comprised an early platelet signal to generate ADP secretion, which was amplified by a secondary thrombin-induced Ca^2+^ signal deriving from SERCA 2b-regulated stores [52]. It was proposed that the thrombin-activated GPCR pathway produced two-second messengers in the platelet system: IP3 mobilized Ca^2+^ from the SERCA 2b Ca^2+^ stores, whereas NAADP released Ca^2+^ as the initial early signal from SERCA 3 Ca^2+^ stores [52]. We find that an apparent similar mechanism may be working in T cells (Figure 4C–E). Figure 4C shows that exposure of Jurkat lymphocytes to tBHQ (2 μM) fails to abolish thrombin responses (0.13 ± 0.03 ratio units, *n* = 5), while also leaving intact subsequent TG responses (0.09 ± 0.006 ratio units) representing the SERCA 2b Ca^2+^ pool. Similarly, the addition of TG (200 pM) first also does not eradicate the thrombin-induced Ca^2+^ release response (0.11 ± 0.008 ratio units, *n* = 5), while subsequent tBHQ responses are also still inducible, albeit significantly reduced (*p* < 0.05, *n* = 5), suggesting that most of the thrombin releasable Ca^2+^ is contained in the SERCA 2b-regulated stores (Figure 4D). Conversely, if T cells are treated first with tBHQ (2 μM) followed by TG (200 pM), thrombin responses are abolished (Figure 4E). This result is consistent with the platelet observations suggesting that thrombin in T lymphocytes appears to release Ca^2+^ from both SERCA 2b and SERCA 3 Ca^2+^ stores.

### 2.4. Despite Differences in SERCA Blocker Sensitivities and Agonist-Mobilizable Ca^2+^ Responses, the SERCA 2b and SERCA 3-Regulated Ca^2+^ Stores Exhibit Similar Ca^2+^ Influx Coupling Actions with Similar Sensitivity to Actin Cytoskeletal Disruption

**Figure 5:**
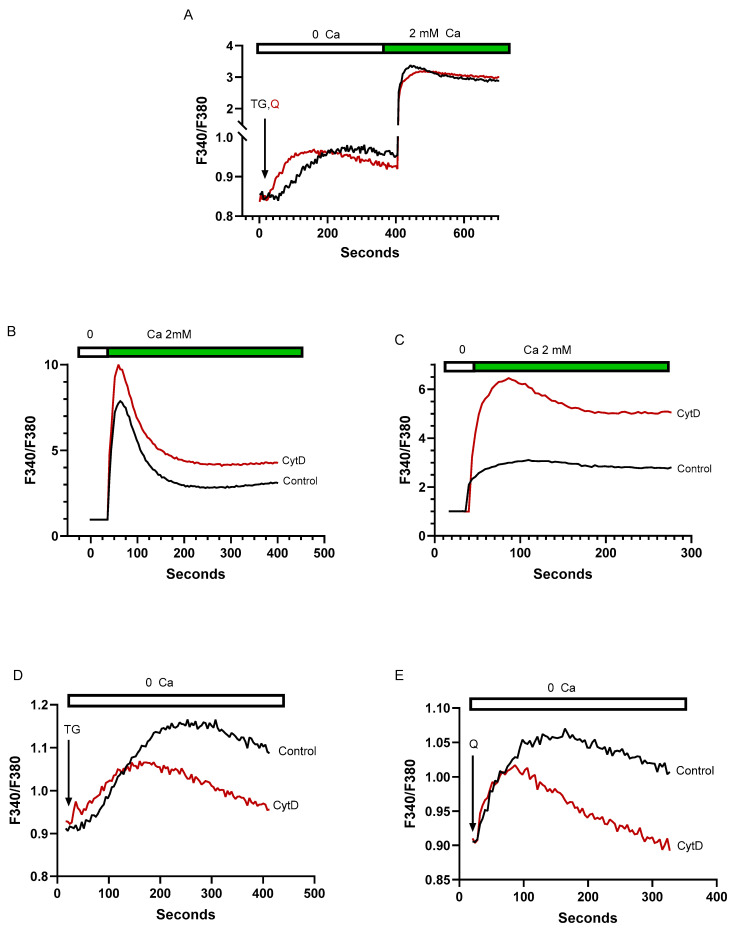
T lymphocyte SERCA 2b and SERCA 3-regulated Ca^2+^ stores reveal similar sensitivities to cytoskeletal disruption with enhanced Ca^2+^ influx responses and reduced Ca^2+^ release activity. For (**A**–**E**), Jurkat T lymphocytes were loaded with Fura-2 and suspended in Ca^2+^-free media (balanced salt solution plus 2 mM EGTA). (**A**). Cells were suspended in Ca^2+^-free conditions (open bar) and stimulated (arrow) with TG (100 pM) or tBHQ (Q, 1 μM). After approximately 400 s of Ca^2+^ release activity, Ca^2+^ levels were increased to 2 mM to elicit Ca^2+^ influx responses (green bar). (**B**), TG, and (**C**), tBHQ-induced Ca^2+^ influx responses in cell populations pre-incubated for 60 min in the presence (red trace) or absence (black trace) of cytochalasin D (10 μM). (**D**,**E**) Jurkat lymphocytes suspended in Ca^2+^-free media were stimulated with TG (D, 200 pM) and tBHQ (E, 2 μM) in the presence (red trace, CytD) or absence (black trace, Control) of cytochalasin D (60 min pre-incubation, 10 μM). Fluorescence traces shown are representative of four to six individual experiments.

T cell activation and gene expression pathways require that SERCA-regulated Ca^2+^ stores communicate with PM Orai channels to mediate Ca^2+^ influx [3,7]. Thus, we sought to investigate whether the TG and tBHQ releasable Ca^2+^ stores described above exhibited similar features in regulating Ca^2+^ influx pathways. The platelet Ca^2+^ signaling system provides a useful framework for understanding the T cell signaling network, which also appears to contain multiple distinct SERCA 2b and SERCA 3 regulated intracellular Ca^2+^ stores. Platelet studies have revealed differences in the SERCA 2b and SERCA 3 regulated Ca^2+^ stores, with the SERCA 3 Ca^2+^ stores demonstrating weaker coupling to Ca^2+^ influx pathways but less sensitivity to cytoskeletal disruption as compared to SERCA 2b Ca^2+^ stores [53]. Our experiments using T cells reveal differences to the platelet system with respect to Ca^2+^ store regulation of Ca^2+^ influx pathways. Figure 5A shows that tBHQ-mediated SERCA 3 blockade and pool depletion activate Ca^2+^ influx responses (2.3 ± 0.52 ratio units, *n* = 12) similar to those induced by TG treatment (2.4 ± 0.71 ratio units, *n* = 15), suggesting that, unlike platelets, the SERCA 3 and SERCA 2b-regulated stores exhibit similar coupling sensitivities to depletion-induced Ca^2+^ influx responses in T lymphocytes. However, in contrast to the platelet system, we find that treating Jurkat cells with the actin cytoskeletal disruptor cytochalasin D (cytD, 10 μM) increases the ability of both SERCA 2b and SERCA 3 Ca^2+^ stores to couple to Ca^2+^ influx responses, as we observed larger Ca^2+^ influx responses in both TG (6.9 ± 2.5 control vs. 9.0 ± 2.1 cytD ratio units, *n* = 4) and tBHQ (2.1 ± 0.88 control vs. 5.5 ± 1.2 cytD ratio, *n* = 4) treated cells (Figure 5B,C). Thus, in T cells, the two Ca^2+^ stores both appear to be negatively regulated by actin polymerization, suggesting that actin dynamics may be interfering with SERCA 2b and SERCA 3 vesicle trafficking to the plasma membrane Ca^2+^ channels. In platelets, the insensitivity of the SERCA 3 Ca^2+^ store to cytD-induced actin disruption led to speculation that this Ca^2+^ pool may reside in close junctional apposition to the plasma membrane [53], which our experiments suggest may not be the case in the T cell system. Intriguingly, we find that pre-treating Jurkat lymphocytes with cytD significantly reduces the TG (0.25 ± 0.03 control vs. 0.14 ± 0.05 cytD ratio units, *p* < 0.05, *n* = 4) and tBHQ (0.16 ± 0.008 vs. 0.10 ± 0.006 cytD ratio units, *p* < 0.05, *n* = 4) inducible Ca^2+^ release responses (Figure 5D,E), suggesting that stable actin filament networks may also be essential for supporting Ca^2+^ release structures, a feature that was not observed in platelet experiments. Indeed, this effect of cytoskeletal perturbation to attenuate Ca^2+^ release may reflect a more depleted Ca^2+^ pool state and thus explain a more robust Ca^2+^ influx coupling response following cytD exposure (Figure 5B,C).

### 2.5. The SERCA Activator CDN1163 Exerts Complex Short- and Long-Term Effects on T Cell Ca^2+^ Stores, Revealing a Differential Regulatory Action on SERCA 2b Versus SERCA 3 Ca^2+^ Pools

We next focused on exploring the effects of the recently identified SERCA activator molecule CDN1163 on T cell Ca^2+^ stores. As mentioned, there is much interest in identifying and characterizing a small-molecule complement to the group of SERCA blockers that can achieve SERCA activation. Our foregoing experiments have further characterized some of the additional complexity in T cell Ca^2+^ stores by revealing distinct SERCA 2b and SERCA 3 Ca^2+^ pools that appear to be recruited to produce Ca^2+^ responses tailored to distinct signaling triggers, including TCR activation, thrombin, and RyR-mediated signals. Thus, given previous work elucidating the salutary effects of CDN1163 attributable to increased activity of SERCA function [8,12,16,26], we anticipated that the compound would produce an augmented Ca^2+^ store condition with perhaps greater Ca^2+^ release responses in the Jurkat T cell system. However, when we measured intact Jurkat cell Ca^2+^ responses treated with varying concentrations of CDN1163, we did not uniformly observe this effect.

**Figure 6:**
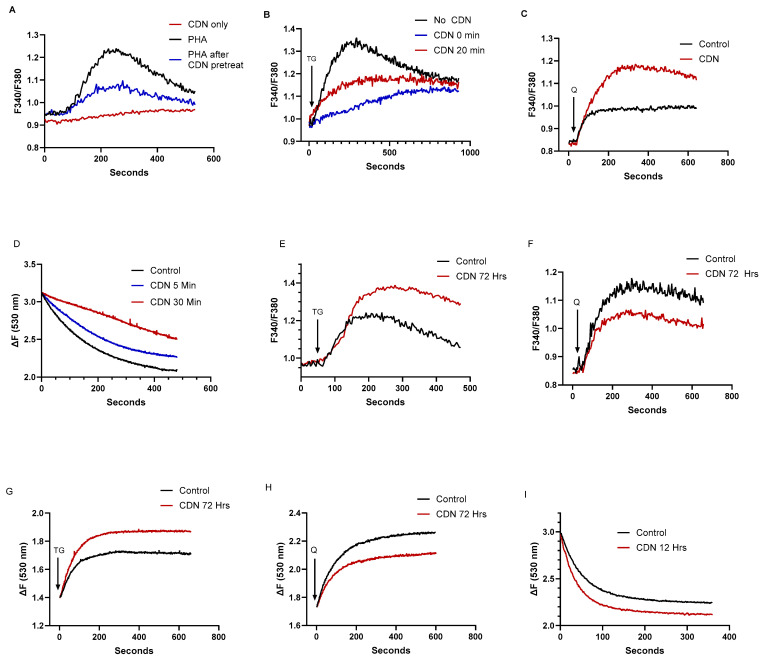
The SERCA activator CDN1163 exerts differential time-dependent effects on T cell SERCA 2b and SERCA 3 Ca^2+^ stores. For A-C Jurkat T lymphocytes were loaded with Fura-2 and suspended in Ca^2+^-free media (balanced salt solution plus 2 mM EGTA). (**A**), Ca^2+^ release responses induced by treatment with PHA (10 μg/mL) in the presence (blue trace) or absence (black trace) of CDN1163 (10 μM, 20 min pre-incubation), also shown is response induced by CDN1163 treatment alone (10 μM, red trace). (**B**), TG (100 pM) induced Ca^2+^ release in the presence (0 min, blue trace and 20 min, red trace, pre-incubation) or absence (black trace) of CDN1163 (10 μM). (**C**), tBHQ (1 μM) induced Ca^2+^ release in the presence (red trace, 20 min pre-incubation) or absence (black trace) of CDN1163 (10 μM). (**D**), Fluo-3 fluorescence Ca^2+^ uptake assay. Ca^2+^ uptake in ER stores was initiated by the addition of ATP (see Section 4) in saponin-permeabilized Jurkat lymphocytes suspended in an intracellular-like medium (ICM, see methods) and incubated in the presence (5 min, blue trace and 30 min, red trace pre-incubation) or absence (black trace) of CDN1163 (25 μM). Rate of Ca^2+^ uptake was estimated based on the linear intial rate of Fluo-3 fluorescence decay. E and F, Ca^2+^ release responses induced by TG and tBHQ in Jurkat lymphocytes incubated for longer durations with CDN1163. (**E**), Ca^2+^ release induced by TG (100 pM) in the presence (red trace, 72 h pre-incubation) or absence (black trace) of CDN1163 (10 μM). (**F**), Ca^2+^ release induced by tBHQ (1 μM) in the presence (red trace, 72 h pre-incubation) or absence (black trace) of CDN1163 (10 μM). G and H show experiments using saponin-permeabilized Jurkat cells, depicting Ca^2+^ release responses as detected by Fuo-3 fluorescence changes. (**G**), Ca^2+^ release induced in permeabilized cells treated with TG (1 nM) in the presence (red trace, 72 h pre-incubation) or absence (black trace) of CDN1163 (10 μM). (**H**), Ca^2+^ release induced in permeabilized cells treated with tBHQ (1 μM) in the presence (red trace, 72 h pre-incubation) or absence (black trace) of CDN1163 (10 μM). (**I**), Ca^2+^ uptake in ER stores was initiated by the addition of ATP (see Section 4) in saponin-permeabilized Jurkat lymphocytes suspended in an intracellular-like medium (ICM, see methods) and incubated in the presence (red trace, 12 h pre-incubation) or absence (black trace) of CDN1163 (25 μM). Rate of Ca^2+^ uptake was estimated based on the linear initial rate of Fluo-3 fluorescence decay. Fluorescence traces shown are representative of six to eight individual experiments for both permeabilized and intact cell assays.

Indeed, Figure 6A shows that a 20 min pre-incubation of T cells with CDN1163 (10 μM) significantly reduced TCR-mediated Ca^2+^ release as measured by PHA treatment in Ca^2+^-free media (peak ratio units: 0.29 ± 0.06 untreated vs. 0.15 ± 0.04 CDN treated, *p* < 0.05, *n* = 7), which represents the IP3 releasable component of the larger TG-sensitive Ca^2+^ stores. We observed the same effect of short-term (<30 min) CDN1163 exposure when cells were treated with low dose TG (100 pM), revealing reduced Ca^2+^ release responses from this pharmacologically defined SERCA 2b Ca^2+^ pool (Figure 6B). Surprisingly, this effect was not observed in the tBHQ-sensitive SERCA 3 Ca^2+^ store. Figure 6C shows that Jurkat lymphocytes exposed to CDN1163 (10 μM) for 20 min exhibit significantly increased Ca^2+^ release responses when treated with low dose tBHQ (peak ratio units: 0.17 ± 0.008 untreated vs. 0.35 ± 0.06 CDN treated, *p* < 0.05, *n* = 8). Thus, our low-dose TG/tBHQ treatment regimen reveals reciprocal effects of short-term CDN1163 exposure on T cell SERCA 2b versus SERCA 3-regulated Ca^2+^ stores. These findings suggest the intriguing possibility that CDN1163 can exert opposing differential regulatory influences on the SERCA pumps, perturbations induced by short-term exposure to the compound that produces diminished Ca^2+^ loading in the SERCA 2b pool with concurrent augmentation of the SERCA 3 Ca^2+^ pool.

Our experiments with CDN1163 in T cells thus yielded somewhat paradoxical effects, given we appear to observe diminished levels of stored Ca^2+^ in the major SERCA 2b-regulated pool after treatment with a putative SERCA pump activator. To further explore CDN1163’s effects on T cell Ca^2+^ stores, we used saponin-permeabilization assays to measure Ca^2+^ uptake responses directly. We incubated Jurkat lymphocytes with CDN1163 (25 μM) over a short time interval (≤30 min) and then proceeded to induce PM permeabilization using low concentrations of saponin. Calcium uptake into ER stores was then initiated by the addition of ATP in a cuvette-based assay, tracking Ca^2+^ uptake into stores by the decline in Fluo-3 fluorescence. Indeed, Figure 6D shows that CDN1163-treated permeabilized Jurkat cells revealed pronounced inhibition of Ca^2+^ uptake as determined by linear initial rates of fluorescence decay (ΔF/s: 5.2 × 10^−3^ untreated vs. 1.5 × 10^−3^ 30 min CDN treated, *n* = 8), suggesting a possible mechanism to explain the observed attenuated Ca^2+^ release responses from the SERCA 2b-regulated Ca^2+^ stores. This result, however, does not explain the apparent augmentation of Ca^2+^ stored in the tBHQ-sensitive SERCA 3-regulated Ca^2+^ pool. It is worth noting that previous studies using Jurkat cells and platelets have revealed that the SERCA 2b-regulated Ca^2+^ store is likely to be larger than the SERCA 3 Ca^2+^ store [27,51,53]. Thus, it is possible that CDN1163 short-term exposure is specifically perturbing the SERCA 2b-mediated Ca^2+^ uptake, which would comprise the dominant effect in our permeabilized cell assays, masking a smaller contribution from increased SERCA 3-mediated Ca^2+^ uptake. We also observed that application of CDN1163 directly to Fura 2-loaded Jurkat lymphocytes induced a gradual, small increase in cytoplasmic Ca^2+^ levels (Figure 6A), further suggesting that the compound over this time interval blocks aggregate SERCA activity and thereby induces Ca^2+^ leakage from the ER Ca^2+^ stores, leading to a relatively depleted state in the SERCA 2b Ca^2+^ store. As above, this result may be explained by the larger effect on the SERCA 2b Ca^2+^ pool experiencing a CDN1163-induced downregulatory action even while the compound exerts a modest increase in Ca^2+^ loading into the SERCA 3 pool. Notably, this ability of CDN1163 to induce gradual increases in cytosolic Ca^2+^ levels with short-term exposure was also recently reported in experiments measuring Ca^2+^ changes in A549 lung epithelial cells [54,55].

Given CDN1163’s well-described action as an allosteric SERCA pump activator [8], we tested whether the compound may require longer periods of exposure to T lymphocytes to increase SERCA activity globally and augment Ca^2+^ store levels in SERCA 2b and SERCA 3 regulated stores. Indeed, we observed that longer exposure to CDN1163 (72 h, Figure 6E) increased Ca^2+^ store levels in the SERCA 2b pool, as revealed by the application of low dose TG (peak ratio units: 0.33 ± 0.06 untreated vs. 0.49 ± 0.07 72 h CDN treated, *p* < 0.05, *n* = 7). Yet, surprisingly, Ca^2+^ store levels in the SERCA 3-regulated stores moved in the opposite direction, exhibiting reduced levels with long-term CDN1163 treatment (Figure 6F), as the low dose tBHQ-induced Ca^2+^ release responses revealed (peak ratio units: 0.54 ± 0.05 untreated vs. 0.39 ± 0.04 72 h CDN treated, *n* = 9). Thus, as with the short-term incubation experiments, we also find in the longer-term exposures a remarkable asymmetry in CDN1163’s effects on the SERCA 2b and SERCA 3-regulated Ca^2+^ stores, with an augmented SERCA 2b and a concomitant reduced SERCA 3 pool with longer CDN1163 treatment. We conducted additional experiments using the permeabilized Jurkat T cell assay to determine if this reversal effect due to longer-term CDN1163 exposure could be observed in direct Ca^2+^ release assays and on Ca^2+^ uptake responses. Indeed, we observed the same pattern in these experiments with permeabilized T lymphocytes as was found using intact cells, detecting increased Ca^2+^ release directly with TG application (Figure 6G) and, conversely, diminished Ca^2+^ release inducible by tBHQ application (Figure 6H) in cells incubated with CDN1163 (10 μM) for 72 h. Intriguingly, it appears that, like TG and tBHQ, CDN1163 exerts differential effects on SERCA 2b and SERCA 3 Ca^2+^ pump isoforms. Thus, our findings suggest that the stimulatory effect of CDN1163 on T cell Ca^2+^ stores is multiplex, requiring prolonged incubation to reverse an initial inhibitory action and augment the SERCA 2b Ca^2+^ store, yet over the extended incubation period the compound exerts a gradual downregulation of Ca^2+^ replenishment of the SERCA 3 regulated store. Using the Ca^2+^ uptake assay in permeabilized cells over a range of different CDN1163 exposure periods, we found that by approximately 12 h Ca^2+^ uptake activity had been restored (Figure 6I), as well as perhaps slightly elevated (ΔF/s: 7.2 × 10^−3^ untreated vs. 8.8 × 10^−3^ CDN 12 h treated, *n* = 8). This time frame for the restoration of ATP-activated Ca^2+^ uptake responses in the presence of CDN1163 aligns well with the general time frame required for the shift in Ca^2+^ release responses to occur in the intact cell experiments.

### 2.6. The SERCA Activator CDN1163 Protects Against TG-Induced Cell Growth Suppression in Jurkat T Lymphocytes but Not Against tBHQ-Induced Growth Inhibition

**Figure 7:**
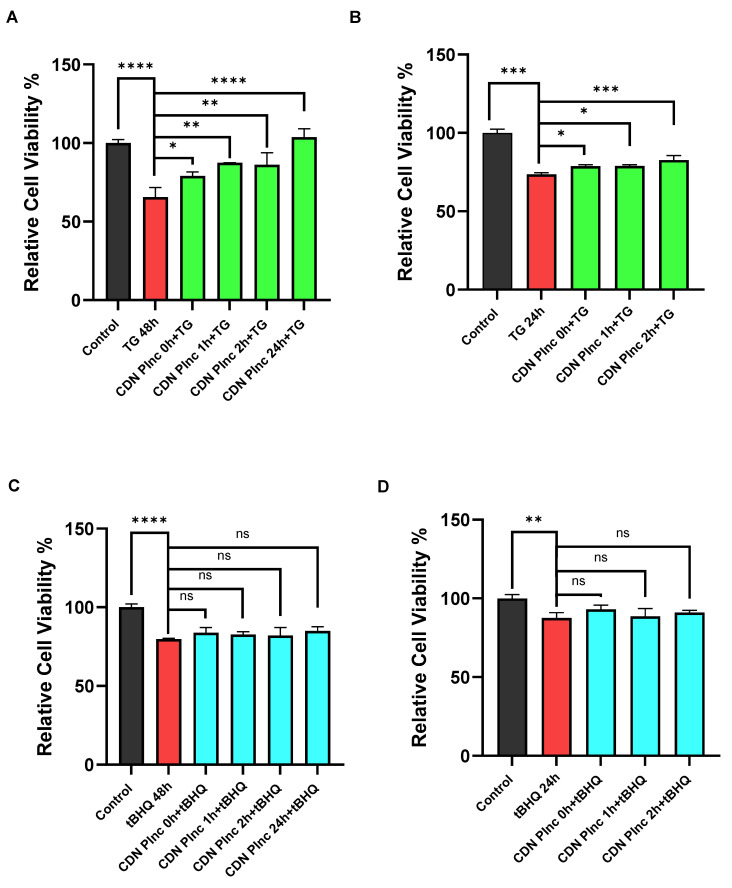
CDN1163 treatment can protect T lymphocytes from thapsigargin but not tBHQ-induced growth inhibition (**A**,**B**), Effect of CDN1163 (10 μM) on TG (1 nM) induced growth inhibition of Jurkat T lymphocytes. Cell growth compared to untreated controls (black bars) was determined after the indicated incubation times and experimental conditions. (**A**). Extending the TG (1 nM) treatment duration to 48 h revealed more substantial effects of CDN1163 in preserving T lymphocyte proliferation as compared to the results in (**B**). Incubation of Jurkat cells with TG (1 nM) for 48 h resulted in pronounced cell growth inhibition (red bar) in the T cell population compared to untreated controls (black bar, *p* < 0.0001). Cells were pre-incubated with CDN1163 (10 μM) for 0–2 h with an additional experiment performed using a more extensive (24 h) pre-incubation with CDN1163 followed by TG (1 nM, 48 h) treatment. Increasing the incubation interval to 48 h revealed greater CDN1163 efficacy (0–2 h) in protecting T lymphocyte growth responses to TG (1 nM)-mediated growth suppression, with the 24 h CDN1163 pre-incubation yielding the most pronounced restoration of growth in cells exposed to TG (1 nM, green bars *p* < 0.0001). (**B**), Incubation of TG (1 nM) for 24 h (red bar) significantly reduced T cell growth compared to the control population (*p* < 0.0001). Pre-incubation (PInc) with CDN1163 (10 μM) for 0, 1 and 2 h followed by TG (1 nM) exposure for 24 h led to increasing protection against TG-induced growth suppression (green bars), with maximal protection in this sequence achieved with a 2 h CDN1163 (10 μM) pre-incubation (*p* < 0.0008). (**C**,**D**), Effect of CDN1163 (10 μM) on tBHQ (2 μM) induced growth inhibition of Jurkat T lymphocytes. (**C**), Extending the tBHQ (2 μM) treatment duration to 48 h resulted in cell growth inhibition (red bar) in the T cell population compared to untreated controls (*p* < 0.0001). Pre-incubation of the cells with CDN1163 (10 μM) for 0–2 h or even 24 h (blue bars) did not significantly protect against tBHQ-induced growth suppression (**D**). Cell growth compared to untreated controls (black bar) was determined after 24 h incubation using the indicated experimental conditions. Incubation of tBHQ (2 μM) for 24 h (red bar) produced modest yet significantly reduced T cell growth compared to control incubation (*p* < 0.0057). Pre-incubation (PInc) with CDN1163 (10 μM) for 0, 1, and 2 h failed to significantly protect T cells in 24 h culture from tBHQ (2 μM)-induced cell growth suppression (blue bars). Asterisks denote statistical significance with * *p* < 0.05, ** *p* < 0.005, *** *p* < 0.001, **** *p* < 0.0001 and ns is not significant *p* > 0.05.

A potential therapeutic value of a SERCA pump activator, as noted in previous work [8,12,16,26], is to achieve robust Ca^2+^ store preservation and potentially protect cells against loss of cell viability due to SERCA dysfunction. In keeping with our experimental results revealing augmented Ca^2+^ store levels secondary to longer-term exposures to CDN1163, we tested whether the compound could preserve lymphocyte viability in cells exposed to TG and tBHQ-mediated SERCA perturbation. Indeed, we found that extended pre-incubation with CDN1163 (10 μM, 24 h) could significantly protect against TG-induced lymphocyte growth suppression (Figure 7A, *p* < 0.0001). Intriguingly, this result corresponds to the same approximate time interval required for CDN1163’s actions to increase Ca^2+^ store release responses that presumably reflect increased Ca^2+^ store levels in the SERCA 2b pools. Shorter-term (0–2 h) pre-incubation of CDN1163 was also able to protect, albeit modestly, against TG (1 nM)-induced growth inhibition with a clear trend to increasing protection efficacy of CDN1163 in longer-term growth intervals (Figure 7A,B). The more limited capacity to protect cell growth during short-interval CDN1163 pre-incubation correlates with our results showing the inability of CDN1163 to increase Ca^2+^ release responses from SERCA 2b pools following shorter-term exposures to the SERCA activator (Figure 6A,B). In contrast, we found that longer-term pre-incubation of T lymphocytes with CDN1163 (10 μM, 24 h) failed to significantly prevent loss of viability to tBHQ (2 μM, Figure 7C), suggesting less involvement of the SERCA 3-regulated pool in influencing T lymphocyte growth responses. Indeed, neither short-term (0–2 h) nor long-term (24 h) pre-incubation with CDN1163 was able to significantly restore lymphocyte growth responses in tBHQ-treated cells in either the 24 or 48 h growth interval (Figure 7C,D). Thus, as with the Ca^2+^ release experiments, we find a differential action of the SERCA activator CDN1163 on the SERCA 2b and SERCA 3-regulated Ca^2+^ stores. It would therefore appear that longer-term exposure to CDN1163 can improve Ca^2+^ loading into the SERCA 2b-regulated store and thereby provide protection against loss of cell viability due to SERCA 2b impairment.

## 3. Discussion

To better assess novel actions of potential SERCA activators within the Ca^2+^ signaling landscape of T cell functions, we were motivated to extend seminal previous work characterizing the major intracellular Ca^2+^ stores. We have employed the strategy of using low concentrations of TG and tBHQ in our experiments, an approach successfully applied to probe the functions of SERCA 2b and SERCA 3-regulated Ca^2+^ pools in human platelets. We applied this strategy using the Jurkat T lymphocyte model, which has been used less extensively than platelets to characterize the properties of the intracellular Ca^2+^ stores. We identified the lowest concentrations of SERCA blockers that elicited measurable Ca^2+^ release responses in cells incubated in Ca^2+^-free media, thus providing greater assurances of using these agents to specifically modulate the SERCA 2b and SERCA 3 pump isoforms. We validated the use of the Jurkat T cell line by verifying that these same effects could be produced in primary lymphocytes isolated from rat splenocytes, confirming that Jurkat cells share the same pharmacological phenotype as primary T lymphocytes when treated with low-dose TG and tBHQ. Indeed, the use of the Jurkat T lymphocyte provides a significant experimental advantage given their clonal homogeneous responses and the ability to cultivate large numbers of cells, features which greatly assist the analysis of measuring relatively weak signals due to modest SERCA perturbations. Moreover, Jurkat lymphocytes continue to be used as powerful T cell model systems given the strong validation and close overlap with primary T cells in the signaling representation of the TCR pathway as the primary upstream activator of ER Ca^2+^ release along with the tightly coupled Ca^2+^ influx pathway [28,30,31,46].

We have examined the Ca^2+^ pool profile in Jurkat lymphocytes with the added discriminatory refinement of employing low concentrations of TG and tBHQ, referencing previous work in human platelets in which this approach has been productively used to gain insight into SERCA 2b and SERCA 3-regulated Ca^2+^ stores [36,39,40,41,51,53]. Using low-dose TG and tBHQ in Ca^2+^-free cell suspensions, we have determined Ca^2+^ release specifically from SERCA 2b versus SERCA 3-regulated Ca^2+^ stores in T lymphocytes. Indeed, our experiments extend earlier investigations to describe at least five distinct Ca^2+^-releasable storage sites in T cells: a TG-sensitive SERCA 2b pool, a subcompartment of the TG-sensitive pool releasable by IP3, a tBHQ-sensitive SERCA 3 pool, a pool dischargeable by agonists of RyRs, a pool releasable by GPCR agonists, and finally the remaining Ca^2+^ storage pool releasable by ionomycin application. These are clearly approximate estimations given agonists, such as thrombin, can release Ca^2+^ from multiple SERCA-controlled stores; and, moreover, Ca^2+^ stores in T cells are likely to contain built-in interconnectivity with Ca^2+^ release from one compartment being captured by a neighboring SERCA-regulated compartment, as has been shown for the RyR-expressing Ca^2+^ pools in T lymphocytes [27,28,43,56]. These observations suggest the intriguing scenario of a complex and dynamic interrelationship among the various intracellular Ca^2+^ stores whereby rapid exchange and flow of Ca^2+^ ions through discrete regional space of the larger ER organelle, whether physically separated or not, may establish de novo spatially localized gradients adapted to accommodate specific T cell signaling functions. These experiments have further characterized the complex array of Ca^2+^ storage compartments and functions in T lymphocytes and have provided a useful foundation of SERCA-specific actions (inducible via low dose TG and tBHQ) to examine the effects of the novel SERCA-activating compound CDN1163.

In contrast to most CDN1163 studies, we did not observe a clear and unambiguous stimulatory effect of the compound on T cell SERCA activity. Indeed, our experiments revealed a surprising short- and long-term acting dichotomy in which an initial period of apparent SERCA inhibition and Ca^2+^ store depletion gradually shifts to SERCA activation and Ca^2+^ store repletion. This discrepancy with previous reports may be due to a more complex SERCA pump expression profile in T cells, given that these cells rely on a minimum of at least two distinct SERCA pump isoforms to manage intracellular Ca^2+^ signaling dynamics. Jurkat T lymphocytes, an often used surrogate for T cell function, are well known for their expression of multiple SERCA isoforms, with the predominant pump species being the SERCA 2b and SERCA 3 isoforms [32,57]. Although the specific protein functions are unknown, Jurkat T cells appear to tap into an extensive diversity in SERCA gene expression, with earlier studies revealing the expression of all six SERCA 3 pump isoforms (SERCA 3a–f) along with the SERCA 2b isoform [35,58], which suggests a high degree of precision and specialized control built in for regulation of T cell Ca^2+^ store functions.

Indeed, some of the apparent incongruous effects we observe with CDN1163 may be due to this complex SERCA environment in T cells, with distinct pump isoforms working in diverse groups of interacting protein partners within heterogeneous ER/PM locales. We find, for example, that when Jurkat lymphocytes are exposed to the putative SERCA activator CDN1163 for short durations (≤30 min), Ca^2+^ release induced by the IP3 pathway or by low-dose TG treatment is significantly reduced. We observed this effect in Ca^2+^ responses measured in both intact and permeabilized cells, a result that paradoxically suggests that CDN1163 may be acting to perturb SERCA function, initiate ER Ca^2+^ leak pathways, and promote loss of ER Ca^2+^ levels. This interpretation is consistent with our experiments using permeabilized cells, in which we observed that short-duration CDN1163 exposure suppressed Ca^2+^ uptake. These actions of CDN1163 to impair ER Ca^2+^ uptake produced a gradual increase in Ca^2+^ release observable in our permeabilized cell assays, which was the likely cause of reduced IP3 and TG-inducible Ca^2+^ release.

Our approach in this study to use low concentration TG and tBHQ to specifically target SERCA 2b and SERCA 3 has provided insight into the novel actions of CDN1163. We report here intriguing differences using these two SERCA blockers in the sensitivity of SERCA 2b and SERCA 3 to the effects of the SERCA activator CDN1163. In our experiments, CDN1163 appears to perturb SERCA 2b-regulated Ca^2+^ stores to a greater extent than the low-dose tBHQ-sensitive SERCA 3 Ca^2+^ store. It is worth noting that CDN1163 has been shown in previous studies to bind to and modulate SERCA 2 isoforms in various cells and tissues, but no clear evidence has emerged for the compound’s effects on the SERCA 3 isoform [8,12,26]. Indeed, we find that CDN1163 attenuates the low-dose tBHQ releasable Ca^2+^ store, albeit with a less pronounced effect as compared to the TG-sensitive pool. Furthermore, we have reported that long-term (>24 h) CDN1163 exposure fails to produce a stimulatory effect with improved Ca^2+^ release inducible by tBHQ, unlike what is observed in the long-term incubation experiments with the TG-releasable Ca^2+^ pool. These findings suggest that there are likely not uniform stimulatory effects induced by CDN1163 across all SERCA pump isoforms, and indeed, these experiments suggest that clear, unambiguous stimulation of SERCA function may be difficult to achieve in T cells and other cells that express multiple SERCA pump isoforms.

Our work does appear to align with previous investigations characterizing CDN1163 as a SERCA activator, albeit acting on an enigmatically slower timeframe in our T lymphocyte model. As mentioned, this effect may be attributable to differential actions of the compound on the different SERCA pump isoforms expressed in the T lymphocyte, with the global cellular SERCA activity being the sum of complex stimulatory and inhibitory effects on SERCA 2b and SERCA 3. Intriguingly, however, CDN1163’s time-dependent augmentation of SERCA 2b Ca^2+^ stores may hint at the compound’s ability to react to and promote differential SERCA states and/or SERCA-pumping environments. Indeed, CDN1163 was initially identified in a chemical library screen for its ability to interfere with SERCA binding interactions with phospholamban [8], the cardiac protein regulator of the SERCA 2a pump isoform. Perhaps CDN1163 is targeting a similar site of regulatory control in the SERCA 2b pump, explaining an initial early period of perturbation on Ca^2+^ transport activity with accompanying Ca^2+^ leak expression; yet with prolonged incubation, regulatory control possibly arising from time-dependent SERCA-associated protein partners re-configures ER systems to enhance Ca^2+^ uptake.

It has been reported that hematopoietic and other cell types can recruit opposing SERCA actions with downregulation of SERCA 3 activity linked to time-dependent upregulation of SERCA 2b expression/function, an observation clearly identified during T cell proliferation and activation [57,59,60]. Our experiments testing the effects of CDN1163 as a SERCA activator on T lymphocyte growth responses align well with these earlier studies. Indeed, we find that treating Jurkat lymphocytes with CDN1163 for a time interval that correlates with the time needed to augment the SERCA 2b Ca^2+^ pool provides significant protection of cell viability to lymphocytes exposed to TG-induced SERCA 2b impairment. Conversely, we did not observe cell protection exerted by CDN1163 to tBHQ-induced growth suppression, which in our experiments is preferentially targeting the SERCA 3 Ca^2+^ pools. Thus, in this study, the ability of CDN1163 to act on SERCA 2b to preserve T lymphocyte growth fits well with previous work revealing preferential isoform upregulation of SERCA 2b expression and activity as an essential underlying cellular response supporting T cell activation and proliferation [57]. Other studies have identified STIM1 as a candidate potential SERCA regulator, such that when ER Ca^2+^ stores experience relative depletion, STIM1, as an ER Ca^2+^ sensor, may participate in SERCA activating functions to replenish ER Ca^2+^ levels [61]. This may also explain the time delay we observe in our T cell model given the initial CDN1163-mediated ER Ca^2+^ leak as a relatively weak, gradual depletion-activating signal that may utlimately couple to STIM1 or other protein regulators to promote greater SERCA activity with increased ER Ca^2+^ transport. Thus, continued interrogation and characterization of CDN1163 may offer an additional SERCA pharmacological tool to probe novel SERCA regulatory networks that appear to play multi-layered Ca^2+^ signaling roles in T lymphocyte signaling.

## 4. Materials and Methods

### 4.1. Materials

Fura 2/AM (fura 2 acetoxymethylester), Fluo-3 pentapotassium salt, pluronic acid, RPMI-160, fetal bovine serum (FBS), streptomycin, and penicillin were obtained from Thermo Fisher. Ryanodine, oligomycin, thapsigargin, and cytochalasin D were obtained from Santa Cruz Biotechnology, Inc. (Dallas, TX, USA). D-myo-Inositol 1,4,5 trisphosphate K salt (IP3), nicotinic acid adenine dinucleotide phosphate sodium salt (NAADP), phytohemagglutinin (PHA), 2,5-di-(*tert* butyl)-1,4-benzohydroquinone (tBHQ), thrombin, creatine phosphokinase (CPK), phosphocreatine disodium salt hydrate, adenosine 5′-triphosphate disodium salt hydrate (ATP), DTT, and saponin were obtained from Sigma. Sterile Cell Strainers (100 μm), 50 mL syringe tubes, and 60 mm cell culture dishes were obtained from Fisher Scientific. CDN1163 was from Bio-Techne (Minneapolis, MN, USA).

### 4.2. Cell Culture

Jurkat cells (Clone E6–1, ATCC TIB-152) were maintained in RPMI-1640 medium supplemented with 10% fetal bovine serum, 2 mM L-glutamine, penicillin (100 IU/mL), and streptomycin (100 μg/mL) and grown at 37 °C in a humidified atmosphere (95% air, 5% CO_2_). Cells were maintained and expanded in either 25 cm^2^ (T25) or 75 cm^2^ (T75) tissue culture flasks (Thermo Fisher Scientific, Waltham, MA, USA). Cell density was not allowed to exceed 3 × 10^6^ cells/mL, and cultures were generally maintained at a cell concentration between 1 × 10^5^ and 1 × 10^6^ viable cells/mL. Fresh medium was added every 2 to 3 days, depending on cell density.

### 4.3. Splenocyte Isolation

The use of animals for these experiments was conducted in accordance with protocols approved by the institutional animal care and use committees at the University of the Pacific (IACUC #22R05). Spleen and lymph nodes were aseptically isolated from adult Sprague-Dawley (SD) rats (8 weeks old, N = 4). Briefly, rats were anesthetized and spleens were aseptically removed and placed into 60-mm cell culture dishes containing ice-cold HBSS (Hanks Balanced Salt Solution, Thermo Fisher Scientific, Waltham, MA, USA) and minced into small pieces with a scissor. Tissue fragments were dissected and passed through a 100-μm cell strainer using a 10 mL syringe plunger and ice-cold HBSS into a 50-mL conical tube and then centrifuged (200× *g* at 4 °C) for 10 min. The supernatant was discarded, and the pellet was resuspended in 5 mL of a red blood cell (RBC) lysis buffer containing 155 mM NH_4_Cl (9 parts) in 130 mM Tris base pH 7.65 (1 part) and incubated at 37 °C for 5 min. RBC lysis was halted by the addition of 10 mL of ice-cold complete cell culture medium, and cells were then centrifuged (200× *g* at 4 °C) for 10 min. The supernatant was discarded, and the pellet was resuspended in 10 mL of complete cell culture medium and maintained in a humidified atmosphere (37 °C, 95% air, and 5% CO_2_).

### 4.4. Cell Calcium Assays

Cells (approximately 1 × 10^6^ cells/mL) were washed in Ca^2+^-containing (1.8 mM) HBSS (Hanks Balanced Salt Solution) and loaded with 1.5 μM fura-2/AM in 20% (*w/v*) Pluronic F-127 and incubated for one hour at 37 °C. After loading, the cells were washed twice with HBSS and incubated at 37 °C for an additional 30 min to allow for de-esterification of the dye. Cells loaded with fura 2/AM were kept in the dark at room temperature throughout the experiments. Changes in cytosolic Ca^2+^ were measured in cell population experiments using a fluorescence spectrophotometer equipped with a thermostatically controlled sample compartment (PTI, Lawrenceville, NJ. USA), permitting continuous stirring of samples in the cuvette. All measurements were carried out at room temperature (25 °C). To achieve Ca^2+^-free conditions, EGTA (2 mM) was added to chelate extracellular Ca^2+^ just before the addition of Ca^2+^ mobilizing agonists (1–2 min). Ca^2+^ changes in Jurkat cells and rat splenocytes loaded with fura 2/AM were measured via rapid alternation of the excitation monochromator between 340 and 380 nm, with fluorescence emission measured at 510 nm using a ratiometric spectrofluorimiter (PTI, Lawrenceville, NJ, USA). Cytosolic Ca^2+^ responses are presented as the changes in the fluorescence ratio values measured at 340/380 nm for Fura-2 or as non-ratiometric Fluo-3 fluorescence changes for the excitation/emission (503/530 nm) wavelength pair. The data are reported as either peak amplitude changes in fluorescence values or as initial rates of fluorescence changes and presented as the means ± S.E.M., with the number of experimental repetitions indicated in parentheses.

### 4.5. Permeabilized Cell Assays

For preparation of permeabilized cells, 4 × 10^7^ cells were washed twice and resuspended in 2 mL of an intracellular-like medium (ICM: 110 mM-KCl, 10 mM-NaCl, 2 mM-MgCl_2_, 20 mM-Hepes, 5 mM-KH_2_PO_4_, pH 7.5) in the presence of 1 mM DTT. Saponin (20 μg/mL) was added, and the cell suspension was incubated for 5 min at 37 °C to complete permeabilization. An ATP-regenerating system consisting of creatine kinase (40 units/mL) and phosphocreatine (20 mM) was added. Oligomycin (10 μg/mL) was also included to inhibit the mitochondrial ATPase. Following cell permeabilization, Fluo-3 (0.5 μM) was added to the cuvette. Subsequent addition of ATP to a final concentration of 1 mM resulted in a decrease in the fluorescence, indicating Ca^2+^ uptake by the intracellular stores. After baseline stabilization, drugs were added according to the experimental plan. Ca^2+^ release from intracellular stores was measured from cells suspended in cuvettes using a fluorescence spectrophotometer equipped with a thermostatically controlled sample compartment maintained at 37 °C with continuous stirring. Fluorescence changes with Fluo-3 in permeabilized cell suspensions were measured with excitation wavelength settings of 503 nm and 530 nm for the emission wavelength.

### 4.6. CRISPR-Cas9 Gene Editing of Jurkat T Lymphocytes

The knockout of SERCA2 (SERCA2_KO_) and SERCA3 (SERCA3_KO_) was carried out using Cas9 protein and single guide RNA (sgRNA) (Thermo Fisher Scientific, Waltham, MA, USA). Cas9 protein (125 μg/mL) and sgRNA (28.2 μg/mL) were combined in Neon NxT resuspension genome editing (GE) buffer and incubated at room temperature to form ribonucleoprotein complexes (Cas9-RNPs). Jurkat T cells, 2 × 10^7^ cells/mL resuspended in GE buffer, were combined with Cas9-RNPs and electroporated with the Neon NxT transfection device (Thermo Fisher Scientific, Waltham, MA, USA) with electroporation parameters (1700 V; 20 ms; 1 pulse). sgRNA sequences were used as follows: (5′-CUUCGGCGUCAACGAGAGUA-3′) for SERCA2 (ATP2A2) and (5′-AGGAUCAGCAUGAUGACCAG-3′) for SERCA3 (ATP2A3), respectively. Electroporated cells were put back into culture for expansion in the RPMI 1640 medium; 72 h post-electroporation, the knockout efficiency was assessed at protein expression level by Western blot, and cells were utilized for cell calcium assays.

### 4.7. Western Blot Analysis

To evaluate gene knockout efficiency, Western blotting was performed using standard methodologies. In brief, after 72 h of Jurkat T cell CRISPR/Cas9 ribonucleoprotein electroporation, cells were collected from culture flasks and pelleted via centrifugation. After washing with ice-cold phosphate-buffered saline, cell pellets were lysed with ice-cold RIPA buffer (Thermo Fisher) supplemented with 1X Halt protease and phosphatase inhibitor (Thermo Fisher Scientific, Waltham, MA, USA) for 20 min on ice. Whole-cell lysates were clarified by centrifugation (15,000× *g* for 15 min at 4 °C), and total protein concentration was determined by the Pierce BCA assay (Thermo Fisher Scientific, Waltham, MA, USA) according to manufacturer protocols. Samples were analyzed using sodium dodecyl sulfate-polyacrylamide gel (SDS-PAGE) electrophoresis and transferred to low-fluorescent PVDF membranes (Bio-Rad, Hercules, CA, USA) by wet transfer. Membranes were blocked for 1 h at room temperature with Intercept Blocking Buffer (LI-COR, Lincoln, NE, USA) and incubated overnight at 4◦C with primary antibodies: anti-SERCA2 (Santa Cruz Biotechnology, Dallas, TX, USA), anti-SERCA3 (Thermo Fisher Scientific, Waltham, MA, USA), anti-ß Actin (Santa Cruz Biotechnology, Dallas, TX, USA). After treatment with primary antibodies, the membranes were washed four times for 5 min with washing buffer (TBST) and incubated with the IRDye 800CW secondary antibodies (LI-COR, Lincoln, NE, USA) for 1 h at room temperature. The bands of proteins were detected by the LI-COR Odyssey M imaging system using Image Studio software Version 6.0. (Lincoln, NE, USA) ß-Actin was used as loading control for all Western blots.

### 4.8. Cell Viability Assay

Lymphocyte viability was measured using the CellTiter 96 AQ_ueous_ Non-Radioactive Cell Proliferation Assay (MTS) system (Promega, Madison, WI, USA) according to the manufacturer’s instructions. Briefly, 3 × 10^4^ cells were plated into each well of a 96-well plate, and at the end of the experiment, 20 μL of MTS reagent was added to the well. After 3.5 h of incubation at 37 °C in a humidified, 5% CO_2_ atmosphere, absorbance at 490 nm was measured using a microplate reader (Spectramax ID3, Molecular Devices, San Jose, CA, USA) to determine the number of cells, which was expressed as a percentage of the control group. Three replicate wells per experimental condition were used to obtain measures of cell *viability*.

### 4.9. Statistical Analysis

For Ca^2+^ measurements in cell populations, responses were compared, and statistical significance was determined using Student’s *t* test. Notably, *p* values ≤ 0.05 were considered to represent significant differences in the results. For lymphocyte proliferation experiments, data were analyzed using one-way ANOVA employing Tukey’s multiple comparisons test, with *p* < 0.05 determining significance.

## 5. Conclusions

We used a well-documented approach in platelets and Jurkat cells of low TG and tBHQ in an effort to specifically modulate SERCA 2b and SERCA 3 regulated T cell Ca^2+^ store functions. Using this approach, we added new insight into complex T cell Ca^2+^ stores. Additionally, we added new detail on the heterogenous profile of intracellular Ca^2+^ stores, noting evidence for at least five distinct Ca^2+^ pools. We observed that a key group of Ca^2+^ mobilizing agonists (TCR, RyR, and GPCR) appear to access SERCA 2b and SERCA 3 in a differential fashion, suggesting SERCA regulation of T cell signaling pathways. We also observed that unlike the platelet system, T cell SERCA 2b and SERCA 3 Ca^2+^ stores appear to be equally sensitive to cytoskeletal disruption, and we examined the actions of the novel SERCA activator CDN1163 on T cell Ca^2+^ store functions. We added new detail to this important new pharmacological tool as the currently best described SERCA activator, showing that the compound appears to regulate T cell SERCA 2b and SERCA 3 Ca^2+^ stores in a complex differential manner, alternately reducing or augmenting Ca^2+^ release functions depending on the length of exposure to the agent. Indeed, we report that CDN1163’s actions to augment the SERCA 2b Ca^2+^ store correlate with the compound’s ability to protect T lymphocyte viability under conditions of TG-induced SERCA impairment. In contrast to previous studies, we suggest that CDN1163 can exert complex effects in cells expressing both SERCA 2b and SERCA 3 pump isoforms that are not unfailingly consistent with pump activation, yet still may provide useful insight into SERCA-regulated Ca^2+^ store functions.

## Data Availability

The original contributions presented in the study are included in the article; further inquiries can be directed to the corresponding author.
